# Diketopyrrolopyrrole (DPP)-Based Materials and Its Applications: A Review

**DOI:** 10.3389/fchem.2020.00679

**Published:** 2020-09-10

**Authors:** Wei Wei Bao, Rui Li, Zhi Cheng Dai, Jian Tang, Xin Shi, Jie Ting Geng, Zhi Feng Deng, Jing Hua

**Affiliations:** ^1^National and Local Joint Engineering Laboratory for Slag Comprehensive Utilization and Environmental Technology, School of Materials Science and Engineering, Shaanxi University of Technology, Hanzhong, China; ^2^Key Laboratory of Rubber–Plastic of Ministry of Education (QUST), School of Polymer Science and Engineering, Qingdao University of Science and Technology, Qingdao, China

**Keywords:** DPP, OFETs, OPVs, sensor, TPA

## Abstract

Diketopyrrolopyrrole (DPP) and its derivatives have been widely studied in the past few years due to its intrinsic physical and chemical properties, such as strong electron-withdrawing, deep color, high charge carrier mobility, strong aggregation, good thermal-/photo-stability. In the 1970s, DPP was developed and used only in inks, paints, and plastics. Later, DPP containing materials were found to have potential other applications, typically in electronic devices, which attracted the attention of scientists. In this feature article, the synthesis pathway of DPP-based materials and their applications in organic field-effect transistors, photovoltaic devices, sensors, two photo-absorption materials, and others are reviewed, and possible future applications are discussed. The review outlines a theoretical scaffold for the development of conjugated DPP-based materials, which have multiple potential applications.

## Introduction

In the past few years, extensive research has developed novel π-conjugated materials, examining different ways to use them in various applications, including organic field-effect transistors (OFET), solar cells, organic light emitting diodes (OLED), coatings, sensors, and so on (Eom et al., 2017; Huang and Li, 2018; Deng et al., 2019; Kwon et al., 2020). These materials offer many technological advantages compared to their inorganic counterparts, such as their low weight, low fabrication cost, foldability, and easy conformation onto non-flat surfaces. Recently, increasing numbers of chemists and physicists have expressed interest in diketopyrrolo-[3,4-c]pyrroloe (DPP) pigments, since DPP-based materials show excellent electronic properties with good thermal and photo-stability (Tieke et al., 2010; Kaur and Choi, 2015).

DPP pigments were commercialized in the 1980s when a crucial structural unit in an important class of red pigments with deep color was first made available. In the beginning, DPP were developed as dyes and pigments and used in inks, paints, and plastics (Iqbal et al., 1988). There were only a few articles on DPP pigments, and the first thiophene-flanked DPP-based polymer semiconductor for OFETs was reported in 2008, which showed hole mobility (μ_h_) of 0.1 cm^2^ V^−1^ s^−1^ and electron mobility (μ_e_) up to 0.09 cm^2^ V^−1^ s^−1^, respectively. Since then, DPP-based conjugated materials have received increasing attention from scientists (Bürgi et al., 2008).

**Figure 1:**
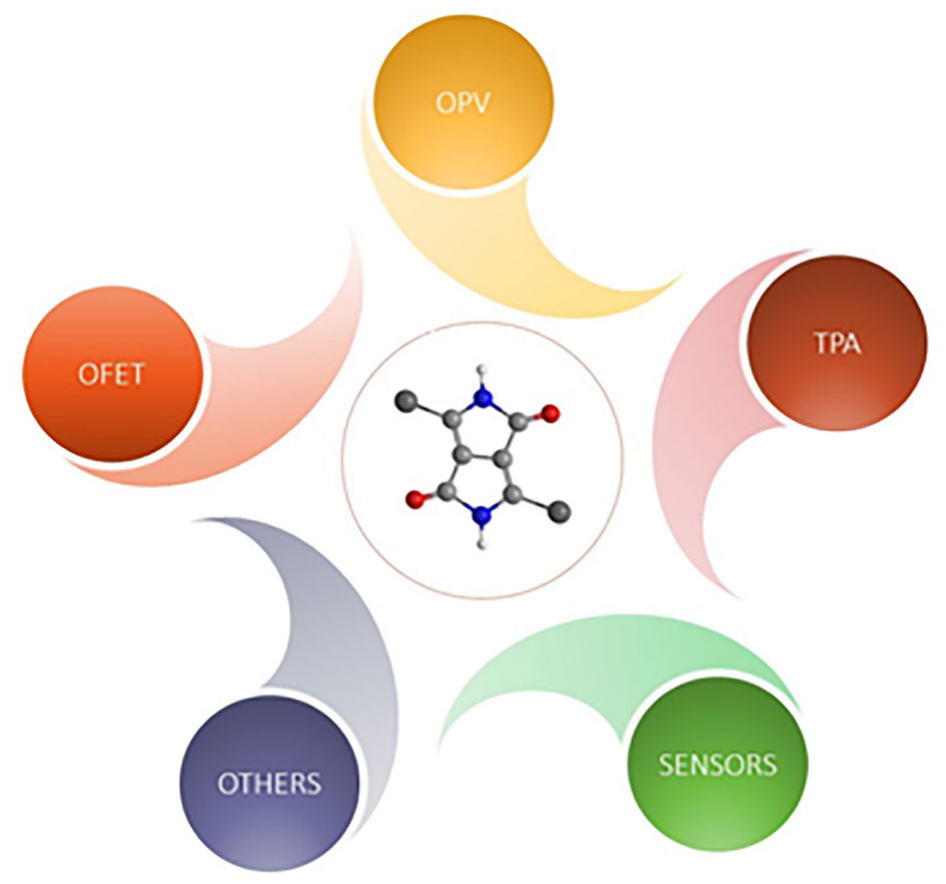
The application of DPP-based materials.

**Figure 2:**
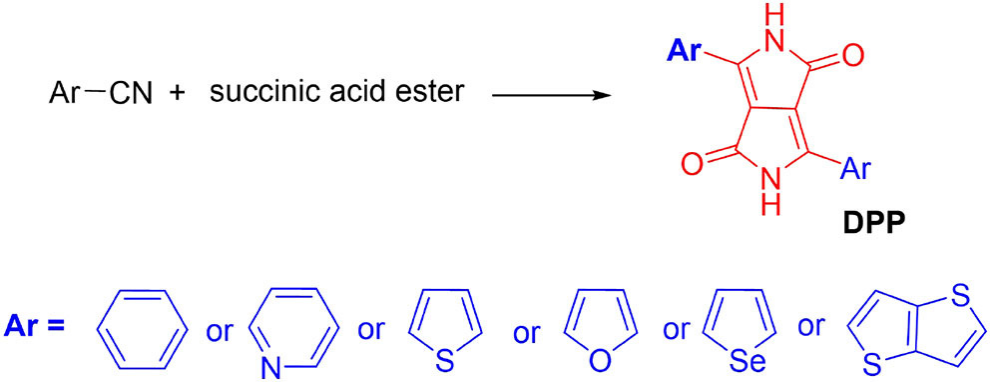
Synthesis of DPP pigments.

DPP pigments were often constructed by a DPP core with two flanked aromatic groups (Figure 2). The core of DPP contains two amine units and carbonyl groups with bicyclic, which endow the DPP pigments with strong electron deficiency properties that can be used for the construction of donor-acceptor (D-A) conjugated materials. DPP-based materials have often exhibited extraordinarily strong π-π interaction and aggregation properties between the neighboring DPP moieties, resulting in the materials having beneficial properties for electronic devices (Qu and Tian, 2012). Till now, the DPP chromophore and its derivatives have played a key role in the molecular design and construction of high-performance materials in electronic devices, including sensors, and so on. Based on the DPP chromophores with numerous advantages, several articles have reviewed its applications in OFETs, sensors, and solar cells separately (Qu and Tian, 2012; Kaur and Choi, 2015; Zhao et al., 2019). However, a comprehensive review of the DPP chromophore and its derivative application has not yet been reported. This article discusses the synthesis pathways, before examining some applications of DPP-based materials, such as OFET, OPV, sensors, two photo absorption materials, coatings, hole transfer materials in perovskite solar cells, etc. (seen in Figure 1), and later, suggests future applications.

## Synthesis of DPP-**Based Materials**

In 1974, Farnum and co-authors first designed and synthesized the DPP pigment flanked with two phenyl units in the low yield (Donald Parnum et al., 1974). The obtained 8-π electron fused ring hydrocarbon pentalene was highly insoluble in most common organic solvents and brilliant red color. Later, Iqbal et al. modified the synthesis pathway by a single reaction step between aromatic nitrile with dialkyl succinate (Figure 2) with a high yield (Iqbal et al., 1988). Subsequently, a large number of DPP derivatives have been designed and reported with the color from red to blue, for example, isomer-DPP, phenyl-/pyridyl-/thienyl-/furanyl-/seleneyl-/thienothiopheneyl- flanked DPP (Figure 2), DPP with distinct alky chain (branch or liner, various length, functionalization, etc.), and so on (Chen et al., 2012; Yiu et al., 2012; Zhang et al., 2013; Yi et al., 2015; Jiang X. et al., 2017; Jiang Z. et al., 2017). These flanked groups could affect the planarity of the DPP pigment, energy levels, and π-π stacking distance. DPP pigments contain two carbonyl and amine units in the core which could form strong hydrogen bonding (NH.OC) in the solid states, resulting in poor solubility in most common organic solvents (Zhang et al., 2017). However, the alkylation could break the hydrogen bonding association, acquiring a good solubility. The soluble DPP with two functional groups, such as bromine, could rend it suitable for polymerization (Tieke et al., 2010). Through the Stille coupling reaction, Yu et al. reported the first DPP-based polymers containing phenyl-flanked DPP and phenylene in the backbone in 1993 (Chan et al., 1993). Later, other approaches including the Suzuki coupling, electrochemical polymerization (Liu et al., 2017), and Buchwald coupling, were developed to synthesize DPP-based polymers (Tieke et al., 2010). Compared with the small molecules or oligomers, D-A typed DPP polymers are more popular in electronic devices. The desirable properties of DPP-based polymers originated from the strong electron deficiency and good planarity of the DPP unit, large π-conjugation system, and remarkable aggregation properties. Further modification of the DPP chromophore is crucial for the application of DPP-based materials.

## Applications

### Organic Field-Effect Transistors (OFETs)

Since the first DPP polymer was reported with the μ_h_ of 0.1 cm^2^ V^−1^ s^−1^ and μ_e_ up to 0.09 cm^2^ V^−1^ s^−1^, the DPP chromophore and its derivatives have obtained a tremendous amount of attention as an electron acceptor in building D-A typed semiconductor materials (Bürgi et al., 2008). DPP-based materials in OFETs can be divided into polymers and small molecule OFETs. Polymers, due to the large π-conjugation system, often show high inter-molecular charge transport mobility. Kim's group have reported a series of DPP-based polymers by varying the ratio between poly [DPP-(*E*)-[2,2-bithiophen]-5-yl)-3-(thiophen-2-yl) acrylonitrile (CNTVT)] unit and DPP-selenophene-vinylene-selenophene (SVS) unit (Khim et al., 2016). In this work, the author found that the charge transport mobility could be effectively modulated from p-channel (μ_h_ = 6.63 cm^2^ V^−1^ s^−1^, μ_e_ = 0.08 cm^2^ V^−1^ s^−1^, CNTVT:SVS = 1:9) to n-channel (μ_e_ = 7.89 cm^2^ V^−1^ s^−1^, μ_h_ = 0.88 cm^2^ V^−1^ s^−1^, CNTVT:SVS = 9:1) dominated by modifying the CNTVT and SVS units. Later, Zhang's groups introduced hydrogen bonding through alkyl-chain engineering to build the OFETs with μ_h_ even up to 12 cm^2^ V^−1^ s^−1^ (Yao et al., 2016). Very soon, Zhang's group investigated thionation isoDPP to isoDTPP could not only improve charge mobility but also result in ambipolar transporting properties (Zhang et al., 2018b).

To the best of our knowledge, the highest charge transfer mobility based on DPP materials was reported by Luo et al. (2016). The author introduced tetramethylammonium iodide into the DPP polymer thin film, which showed the μ_h_ up to 26 cm^2^ V^−1^ s^−1^ and μ_e_ to 4.4 cm^2^ V^−1^ s^−1^. Such high charge carrier mobilities are ascribed to the more ordered lamellar packing of the alkyl side chains and inter-chain π-π interactions. For small molecular OFETs, since the molecular packing with more crystalline can be easily modified by the chemical structure, they often showed good intra-molecular charge transport ability. Recently, hydrogen bonding association in DPP-based materials enhanced the charge carrier mobility, as reported by Zhang and other groups (Oh et al., 2016; Zhang et al., 2017, 2020). Through hydrogen bonding association, the crystal-to-crystal transition was studied in crystal DPP-based semiconductors by Zhang et al. (2017). Even the hole mobility was only 0.26 cm^2^ V^−1^ s^−1^, this work provided a simple and useful approach to obtain high cystallinity OFET devices with insoluble pigment molecules through their soluble precursors.

Compared with other types of organic material semiconductors, more DPP-based materials were reported with high charge carrier mobility, typically over 1 cm^2^ V^−1^ s^−1^. DPP-based materials show high charge carrier mobilities. This is probably due to its strong electron-deficient ability, good planarity, and strong aggregation, which cause the materials to have good inter-/intra- charge transport ability within the semiconductor layer. Design of DPP chromophore, such as modification with the flanked aromatic groups, alkyl chain, and the core of the DPP, etc., is crucial for achieving good semiconductor materials.

### Organic Photovoltaics (OPVs)

Among the various renewable energy sources, solar energy, renewable, and carbon-neutral energy sources are unarguably the most exploitable sources. More solar energy reaches our planet in 1 h that is consumed by mankind in an entire year (Hammarström and Hammes-Schiffer, 2009). Thus, photovoltaic cells, which directly convert solar energy to electrical energy, represent the most promising renewable energy device. Up to date, the organic solar cell (OSCs) has received a large amount of attention due to its promising performance, its simple solution-processable fabrication technique, low cost, and sustainability (Chen et al., 2020; He et al., 2020). Recently, more scientists have turned their attention to chemical structure modification, regarding organic materials. Among these, the DPP pigment has a high profile and often plays an important role in the molecular design concept for high-performance materials in OSCs.

In 2008, Janssen's group reported a polymer containing thiophene-flanked DPP and bithiophene and used it to construct bulk-heterojunction (BHJ) solar cells with C_60_ and PC_70_BM, which showed a power conversion efficiency (PCE) up to 4.0% with open-circuit voltage (V_oc_) of 0.61 V (Wienk et al., 2008). In this OSC, the DPP-based polymer operated as a donor and fullerene worked as an electron acceptor. Later, the same group modified the alkyl chain and donor unit (phenyl instead of bithiophene) for polymerization. The obtained polymer exhibited slightly higher PCE ( 5.5%) and higher V_oc_ ( 0.8 V) (Bijleveld et al., 2010). The higher V_oc_ could be ascribed to: (i) in most cases, the V_oc_ in BHJ solar cells are related to the energy difference between the lowest unoccupied molecular orbital (LUMO) levels of the acceptor and the highest occupied molecular orbital (HOMO) levels of the donor (conjugated materials); (ii) the donor units in the materials mainly contribute to the HOMO levels; (iii) phenyl showed weak donor ability compared to thiophene. This also has been studied in the DPP-based oligomers (Qu et al., 2012).

Compared with the fullerence OSCs, the non-fullerence (NF) OSCs are more popular due to various advantages such as tunable absorption, structure versatility, energy levels, and crystalline (Qu and Tian, 2012; Gao et al., 2019; Zhao et al., 2019). DPP-based materials in OSCs belong to state-of-the-art materials in NFOSCs, due to its strong electron-withdrawing ability and broad optical absorption even into the near-infrared (NIR) region. Li's group reported DPP-based polymers containing Th-DPP and oligothiophene segments (Jiang X. et al., 2017). These polymers showed strong absorption between 500 and 1,000 nm, which match well with the solar spectrum. Using these polymers as electron donor and 3,9-bis(2-methylene-(3-(1,1- dicyanomethylene)-indanone))-5,5,11,11-tetrakis(4-hexylphenyl)-dithieno[2,3-d:2',3'-d']-s-indaceno[1,2-b:5,6-b']dithiophene (ITIC) as electron acceptor to build NFOSCs resulted in the PCE of 1.9–4.1%. These studies indicated that it is crucial to tune the miscibility between donor and acceptor for improving the performance of NFOSCs. Compared to polymers, small molecules and oligomers are purified and modify the chemical structure more easily. In 2019, Gao et al. reported two NIR absorbing DPP-based oligomers and used them as acceptors combined with 6TIC as a donor to construct NFSOCs (Gao et al., 2019). To the best of our knowledge, these NFSOCs showed the best performance among the DPP-based solar cells with the PCE up to 12.08%. The high device performance could be ascribed not only to the chemical and physical properties of the oligomer structure, but also the good film morphology. DPP-based conjugated materials, typically the thiophene-flanked DPP, often showed broad absorption spectra with NIR tail, high charge carrier mobility, and crystallinity, thus it is widely used in OSCs. Further functionalization of DPP materials, such as furanone-/seleneyl-flanked DPP or isoDPP, combined with optimizing device fabrication, might be critical in realizing high performance OSCs.

### Sensors

A chromophore bonded with a specific analyte can cause either an increase or decrease in the emission/absorption intensity, accompanied by the phenomenon of a red or blue shift of the emission band or absorption band. These kinds of chromophores were widely used as sensors (Kaur and Choi, 2015). Recently, DPP molecules have been popular in ions sensors because of functional units such as carbonyl and amine groups. In 2018, Zhang et al. reported that a DPP-based polymer film contains lactam amide units. This work firstly indicates that the DPP-based polymer film can be used not only as a renewable fluoride anion chemosensor with detection limits as low as 10^−6^–10^−8^ M, but also a promising fluoride anion extractor (Zhang et al., 2018a). Jang's group reported the DPP small molecule for detection cyanide anion due to the functional carbonyl units of the DPP core (Jeong et al., 2012). The original DPP small molecule in the solution shows green color with strong emission. However, once the cyanide anion was added, the solution color changed into red with quenching fluorescence.

Through molecular design, DPP-based materials with NIR absorption and emission are popular in cell sensors. This could be attributed to the fact that the low energy from the NIR light can minimize photo-damage to biological cells and deeply penetrate tissue for cell recognition (Kaur and Choi, 2015). Wiktorowski et al. reported water-soluble DPP derivatives with NIR fluorescence and internalized it into live-cell images of CHO cells using a confocal fluorescence microscope (Wiktorowski et al., 2014). The result showed that the intra-cellular fluorescence quantum yield (ϕ_f_) of the DPP derivative was 34%. Compared with the most used indocyanine green, DPP derivatives showed not only high intra-cellular ϕ_f_ but also improved intra-cellular photostability. Further modification DPP derivatives, typically focusing on good photostability, high ϕ_f_, water solubility, along with two-photo absorption and emission, is crucial for the development cell or DNA sensors.

### Two-Photon Absorption (TPA)

Conjugated molecules exhibiting large TPA cross sections (δ) can be applied in the photo-sciences, for example in two-photon fluorescence imaging, optical power limiting, two-photon up conversion lasing, three-dimensional optical data storage and so on (Xu et al., 2020). Although there are numerous conjugated materials with TPA properties, few among them exhibit large δ. In 2008, Yang's group reported that the phenyl amine-DPPs small molecule showed large δ up to 1,200 GM (Guo et al., 2009). Later, this group studied the DPP-based polymer, which also displayed high δ (859 GM) (Zhang et al., 2011). Since then, the DPP chromophore was widely studied and used in the design of TPA materials. Ftouni et al. reported a series of DPP-based small molecules with δ being 750 GM, and successfully coupled these molecules to a synthetic Tat-derived peptide as a two-photon fluorescent tag for living cell microscopy with low power excitation (Ftouni et al., 2013). Later, Hua's group reported DPP oligomer with not only large δ (1,140 GM) and high fluorescence brightness after aggregation (39.02%), but also strong aggregation-induced emission properties (Jiang et al., 2016). These kinds of materials are promising in terms of bio-application, except for bio-imaging, and DPP materials with TPA properties used in heavy metal sensors, such as Hg^+^, as reported by Nie and co-authors (Nie et al., 2018). Till now, the DPP-based TPA materials have a large space and research has explored multi-photon absorption, but examinations of three-photo absorption are quite rare. In 2017, Ye et al. firstly reported a series of DPP oligomers with three-photon absorption through computation (Ye et al., 2017). Unfortunately, these materials have not been synthesized, but through the development of molecular design concepts, multi-photon absorption materials based on DPP derivatives are on the way.

### Other Applications

With the exception of the application described above, the intrinsic physical and chemical properties of DPP-based materials mean they are were widely used in other applications. Reversible electrochromism properties regarding DPP-based polymers were reported by Tieke's group (Zhang et al., 2013). These studies reveal that DPP-based materials had potential in color-change windows. In the beginning, DPP pigment was developed as a dye in inks and paints due to its weather resistance and deep color. Based on these properties, Zhang and Zhou et al. introduced DPP into the one-coat epoxy coating, which showed improvement in UV-stability compared to the pure epoxy coating, due to the DPP pigment creating strong UV-light absorption (Zeng et al., 2018). DPP chromophore, typically the thiophene-flanked DPP with high hole transfer mobility, were used in dopant-free hole transfer materials (HTM) in perovskite solar cells, which not only improved the device stability but also reduced the cost of HTMs compared to the state-of-art HTMs (spiro-OMeTAD) (Li et al., 2019; Zhang et al., 2019; Chang and Wang, 2020). Data's group reported phenyl-, furanyl- and thienyl-flanked DPP as emitters in OLED devices, which showed the EQE up to 12.1% (Data et al., 2016).

## Conclusion and Outlook

In summary, we have provided an overview of the synthesis and applications of DPP-based π-conjugated materials. Various aromatic-flanked DPP derivatives were described. DPPs are insoluble pigment due to the strong hydrogen bonding, which through alkylation of the amide units and bromination of the aromatic units could be converted into soluble monomers for polymerization. DPP chromophore exhibits strong electron-deficient properties, good planarity backbone, and strong aggregation in the solid-state. It has been widely used in the construction of D-A typed high performance semiconductor materials. Due to its broad optical absorption combined with high charge transfer mobility and good photo-/thermal-stability, DPP-based materials were widely used in solar cells, typically non-fullerence solar cells. The carbonyl and amine groups in the DPP core lead to DPP derivatives suitable for many ion sensors and cell sensors, particularly in the NIR region sensor and TPA biological sensor. Moreover, the application of DPP in other fields, such as coating, chemical color change window, photo-detector, OLED, etc., would also benefit from exploration in the future.

Compared with the phenyl- and thienyl-flanked DPP, the seleneyl- and pyridyl-flanked DPP are less studied. The pyrridyl-flanked DPP might display low LUMO levels with good electron transfer mobility. The seleneyl-flanked DPP might show improved planarity and good aggregation, thus high charge transfer mobility is expected. It would be worthwhile to further functionalize for DPP and its derivatives, such as enlargement of π-conjugation, modification of alkyl chain, design of high-performance polymers for the promotion in π-conjugated materials applications. DPP-based conjugated materials might play a key role in realizing high performance electronics as well as other applications, thus further design of DPP-based materials and development of its applications are required.

## Author Contributions

WB and RL collected the articles and wrote the first manuscript. ZD and XS collected the articles and modified the format. JT organized references and revised the manuscript. JG wrote the part of Other applications. ZD revise the manuscript and approved the final version. JH conceived of the topic and supervised the whole work. All authors contributed to revise the manuscript, approved the final version and agreed to be accountable for all aspects of this work.

## Conflict of Interest

The authors declare that the research was conducted in the absence of any commercial or financial relationships that could be construed as a potential conflict of interest.
